# Supplementary material for: Ligand-induced activation and G protein coupling of prostaglandin F2α receptor
Source: Nat Commun. 2023 May 9;14:2668. doi: 10.1038/s41467-023-38411-x (PMC10169810; doi:10.1038/s41467-023-38411-x)
Supplement: Supplementary file 1 — Supplementary Information [file 41467_2023_38411_MOESM1_ESM.pdf]

Ligand-induced activation and G protein coupling of prostaglandin $F_{2\alpha}$ receptor

Canrong Wu[1,*,#], Youwei Xu[1,*], Qian He[1], Dianrong Li[2], Jia Duan[1], Changyao Li[3,4], Chongzhao You[1], Han Chen[5], Weiliang Fan[2], Yi Jiang[3,4], H. Eric Xu[1,6,#].

[1]State Key Laboratory of Drug Research, Shanghai Institute of Materia Medica, Chinese Academy of Sciences, Shanghai 201203, China

[2]Sironax (Beijing) Co.,Ltd. Beijing 102206, China

[3]Lingang laboratory, Shanghai 200031, China

[4]School of Life Science and Technology, ShanghaiTech University, 201210 Shanghai, China

[5]Department of Biochemistry and Molecular Biology, School of Basic Medical Sciences, Fujian Medical University, Fuzhou, Fujian, 350108, China.

[6] University of Chinese Academy of Sciences, Beijing 100049, China

[#]Co-corresponding authors

H.E.X., email: eric.xu@simm.ac.cn,

C. W.: wucanrong@simm.ac.cn

*These authors contributed equally to this work.

Supplementary Figure 1

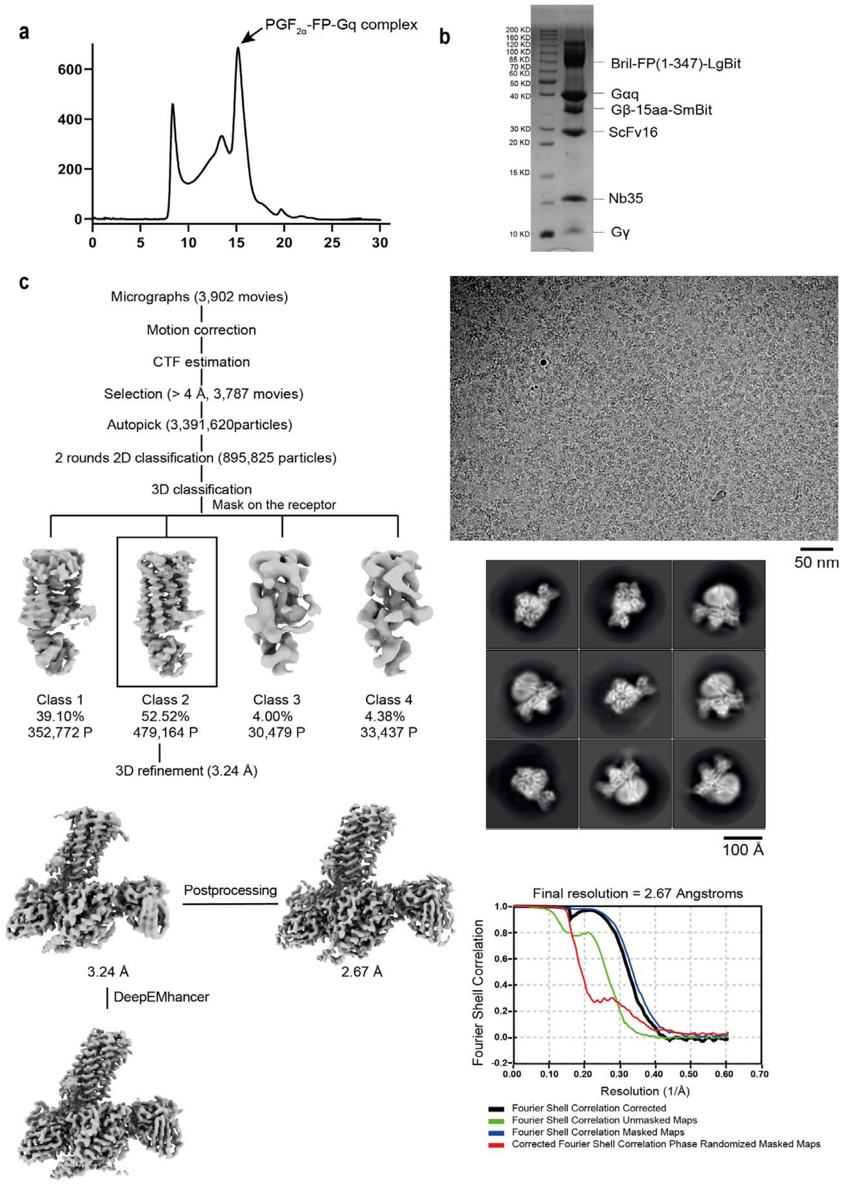

Supplementary Figure 1. PGF$_{2\alpha}$-FP-G$_q$ complex purification and cryo-EM data processing. a,b, Representative size-exclusion chromatography elution profile (a) and SDS-PAGE analysis (b) of PGF$_{2\alpha}$-FP-G$_q$ complex. Black arrow refers to complex monomer. The cryo-EM sample preparation and data collection were performed once. c, Computational sorting of cryo-EM particle images, the “Gold-standard” FSC curve. In panels a-b, the experiments were repeated independently at least three times with similar results. Source data are provided as a Source Data file. Uncropped scan of gel in panel b is provided at the end of the Supplementary Information file.

Supplementary Figure 2

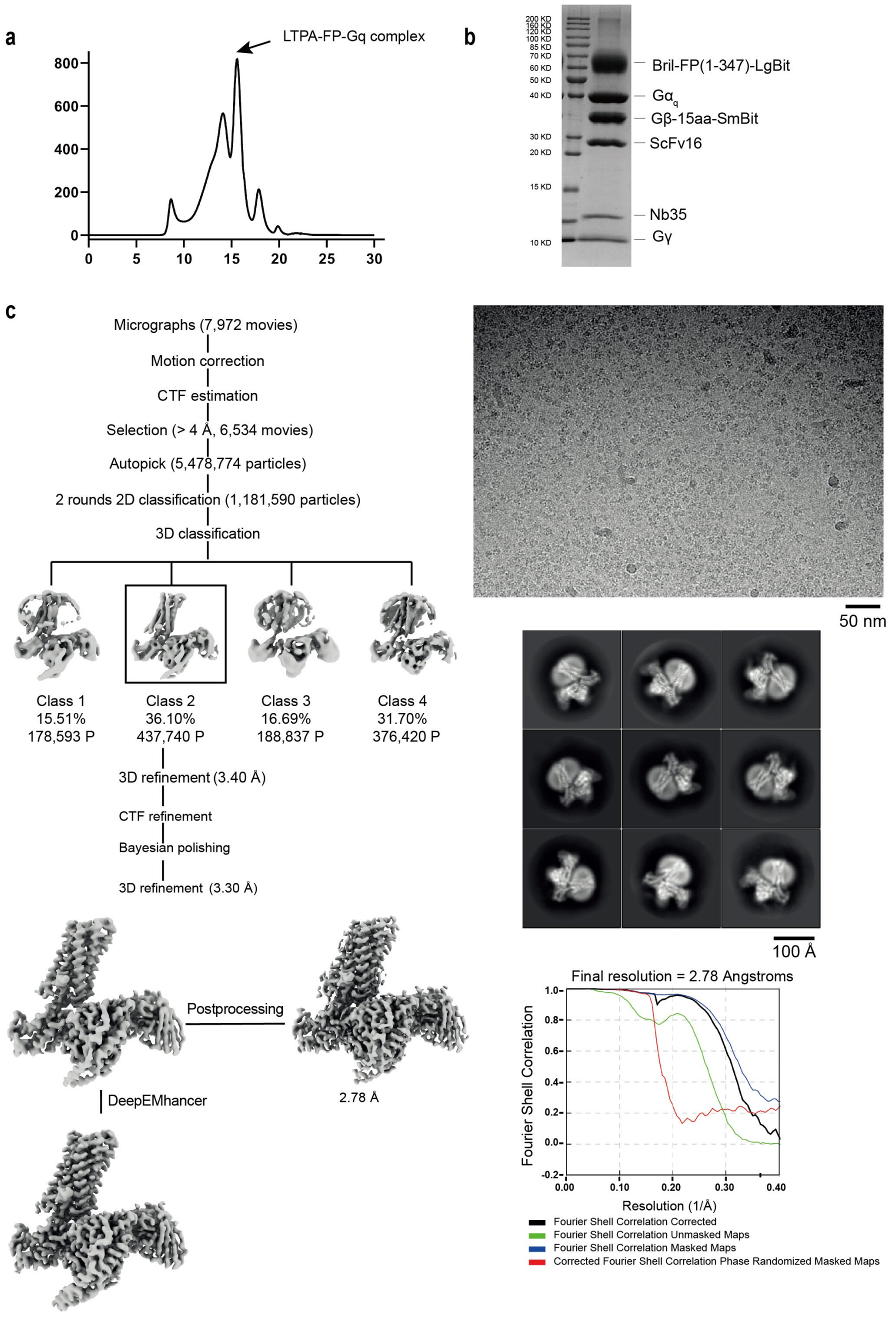

Supplementary Figure 2. LTPA-FP-G_q complex purification and cryo-EM data processing. a,b, Representative size-exclusion chromatography elution profile (a) and SDS-PAGE analysis (b) of LTPA-FP-G_q complex. Black arrow refers to complex monomer. The cryo-EM sample preparation and data collection were performed once. c, Computational sorting of cryo-EM particle images, the "Gold-standard" FSC curve. In panels a-b, the experiments were repeated independently at least three times with

similar results. The cryo-EM sample preparation and data collection were performed once. Source data are provided as a Source Data file. Uncropped scan of gel in panel b is provided at the end of the Supplementary Information file.

Supplementary Figure 3

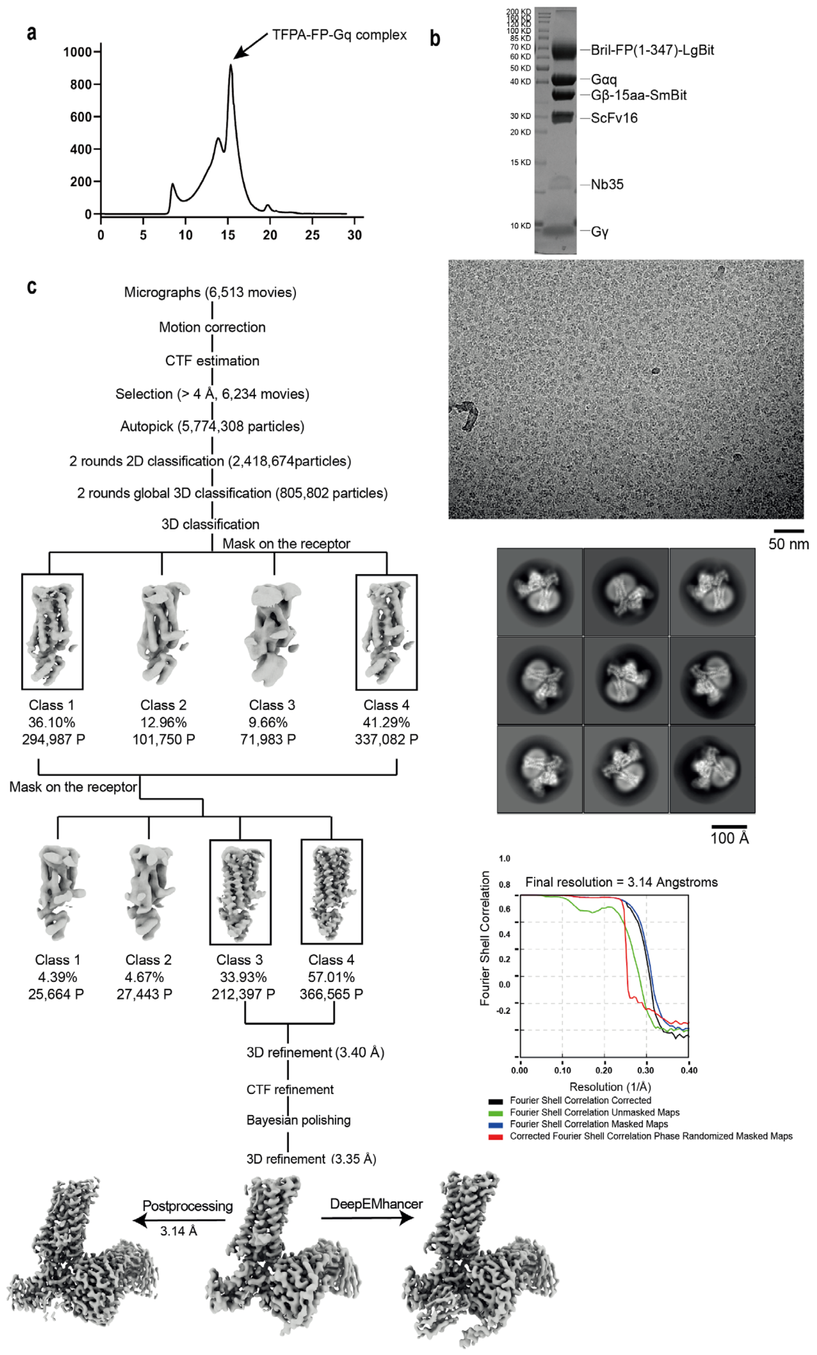

Supplementary Figure 3. TFPA-FP-G_q complex purification and cryo-EM data processing. a,b, Representative size-exclusion chromatography elution profile (a) and SDS-PAGE analysis (b) of TFPA-FP-G_q complex. Black arrow refers to complex monomer. The cryo-EM sample preparation and data collection were performed once. c, Computational sorting of cryo-EM particle images, the "Gold-standard" FSC curve. In panels a-b, the experiments were repeated independently at least three times with

similar results. The cryo-EM sample preparation and data collection were performed once. Source data are provided as a Source Data file. Uncropped scan of gel in panel b is provided at the end of the Supplementary Information file.

Supplementary Figure 4

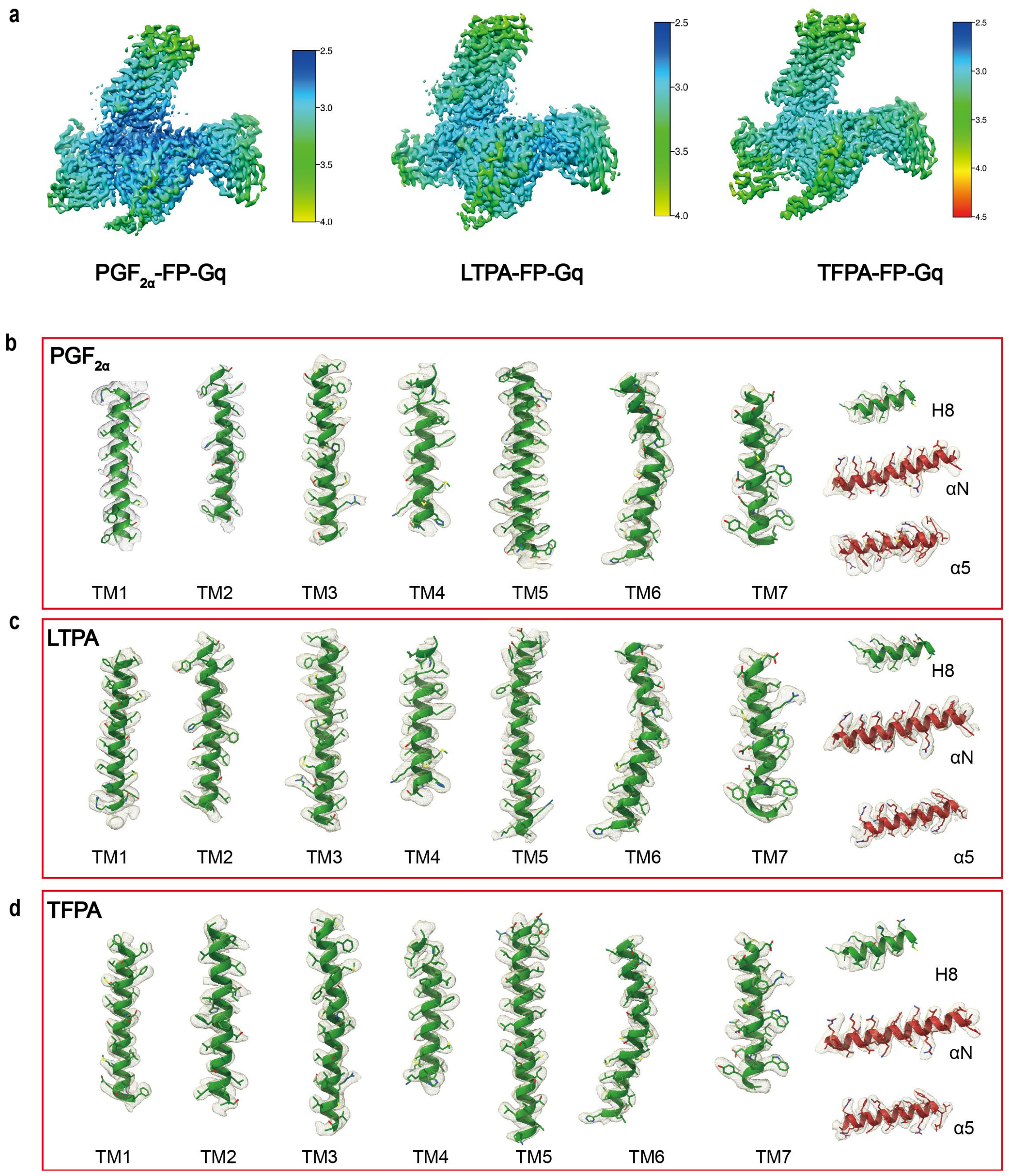

Supplementary Figure 4. Representative cryo-EM density maps of the $PGF_{2\alpha}$/ LTPA/ TFPA-FP-G_q complexes. a, The local resolution map of $PGF_{2\alpha}$/ LTPA/ TFPA-FP-G_q

complexes. b-d, Cryo-EM density maps of the seven transmembrane (TM) helices, α5 helix of Gα_q and αN helix of Gα_q for PGF$_{2\alpha}$ bound FP (b), LTPA -bound FP (c), and TFPA-bound FP (d) were shown.

Supplementary Figure 5

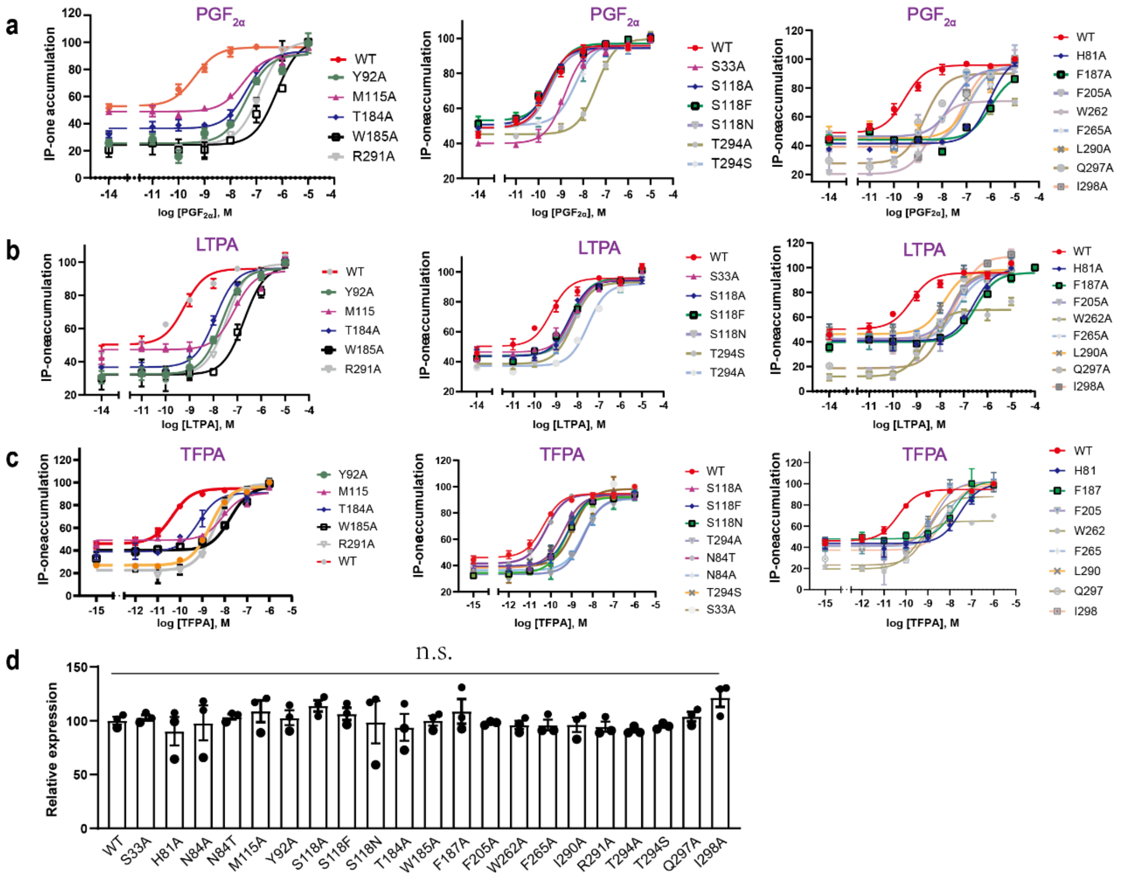

Supplementary Figure 5. Effects of mutation in the ligand binding pocket of FP on IP-One accumulation. (a-c), PGF$_{2\alpha}$, LTPA, and TFPA response curves on WT and mutant FP receptors; (d), Cell surface expression level of WT and mutant FP receptors. Data are presented as mean values ± SEM; n=3 independent samples, significance was determined with two-side unpaired t test; P >0.05 was considered statistically no significant (n.s.). Exact P values and Source data are provided as a Source Data file.

Supplementary Figure 6

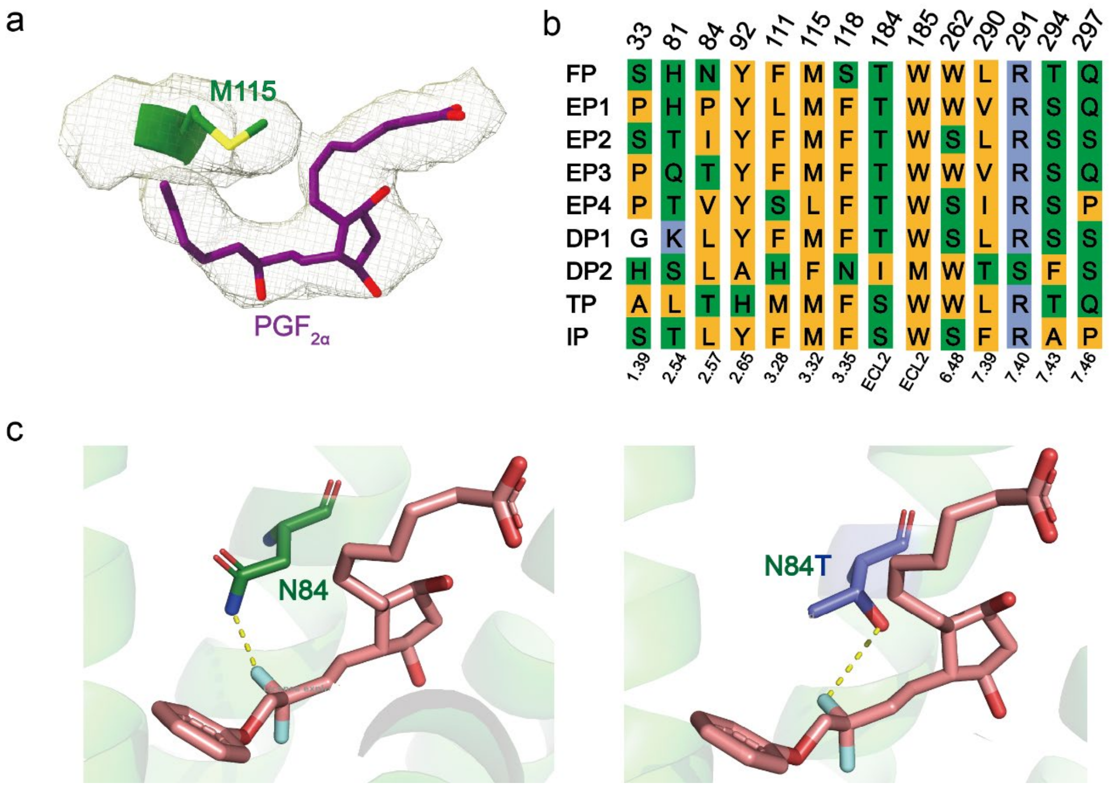

Supplementary Figure 6. Ligand interaction of FP. a, the interaction of M115 in FP with $PGF_{2\alpha}$. Residue and ligand are shown in sticks, with the correspondent cryo-EM density represented in mesh. b, Sequence alignment of prostonoid receptors. Only residues in $PGF_{2\alpha}$ binding pocket are displayed. c, The detailed interactions of TFPA with N84 of FP and T84 of mutant FP.

Supplementary Figure 7

a

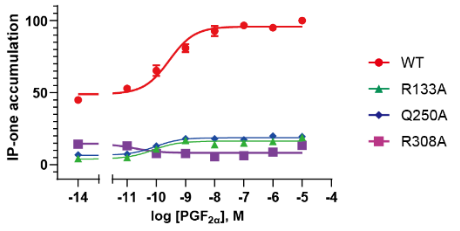

	pEC$_{50}$	E$_{min}$	E$_{max}$
WT	9.6 ± 0.3	48.9 ± 5.0	95.8 ± 3.2
R133A	10.1 ± 0.6	4.0 ± 2.8	16.4 ± 1.5
Q250A	10.0 ± 0.6	6.5 ± 0.8	18.8 ± 1.1
R308A	NA	8.2 ± 1.7	14.5 ± 3.3

b

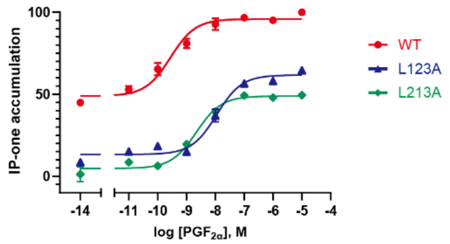

	pEC$_{50}$	E$_{min}$	E$_{max}$
WT	9.6 ± 0.3	48.9 ± 5.0	95.8 ± 3.2
L123A	8.0 ± 0.2	13.3 ± 2.7	61.7 ± 4.1
L213A	8.7 ± 0.2	4.8 ± 1.9	48.8 ± 2.6

c

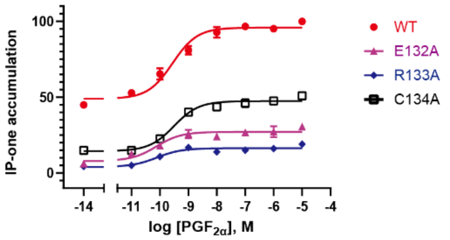

	pEC$_{50}$	E$_{min}$	E$_{max}$
WT	9.6 ± 0.3	48.9 ± 5.0	95.8 ± 3.2
E132A	10.1 ± 0.6	8.0 ± 4.3	27.1 ± 2.2
R133A	10.1± 0.6	4.0 ± 2.8	16.4 ± 1.5
C134A	9.5 ± 0.3	14.5 ± 3.3	47.4 ± 1.9

d

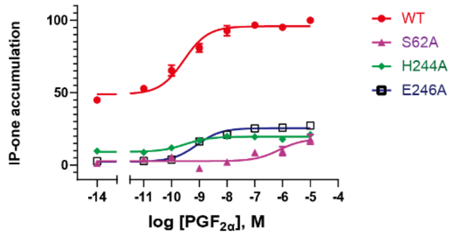

	pEC$_{50}$	E$_{min}$	E$_{max}$
WT	9.6 ± 0.3	48.9 ± 5.0	95.8 ± 3.2
S62A	/	2.7 ± 2.3	18.3 ± 7.1
H244A	9.5 ± 0.6	9.2 ± 2.5	19.7 ± 1.5
E246A	9.1 ± 0.2	2.4 ± 1.4	25.6 ± 1.1

e

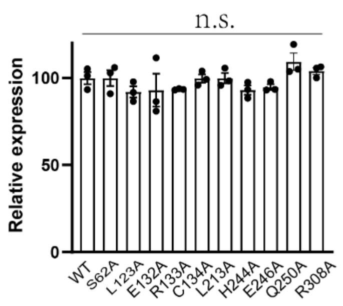

f

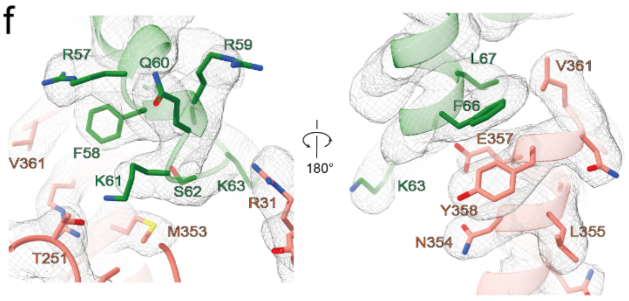

Supplementary Figure 7. Effects of mutation in the activation and Gq coupling of FP on IP-One accumulation. (a-d), PGF$_{2\alpha}$ response curves on WT and mutant FP receptors; (e), Cell surface expression level of WT and mutant FP receptors. Data are presented as mean values ± SD; n=3 independent samples, significance was determined with two-side unpaired t test; P >0.05 was considered statistically no significant (n.s.). Exact P

values and Source data are provided as a Source Data file. (f) The detailed interactions of TM1, TM2 and ICL1 with the αN and α5 helices of Gαq. Source data are provided as a Source Data file.

Supplementary Table 1

Cryo-EM data collection, model refinement and validation statistics.

	$PGF_{2\alpha}$-FP-G_q complex	LTPA-FP-G_q complex	TFPA-FP-G_q complex
**Data collection and processing**			
Magnification	105,000	105,000	105,000
Voltage (kV)	300	300	300
Electron exposure ($e^-/Å^2$)	50	50	50
Defocus range (μm)	-1.0~-3.0	-1.0~-3.0	-1.0~-3.0
Pixel size (Å)	0.824	0.824	0.824
Symmetry imposed	C1	C1	C1
Initial particle projections (no.)	3,391,620	5,478,774	5,774,308
Final particle projections (no.)	479,164	437,740	578,962
Map resolution (Å)	2.67	2.78	3.14
Map resolution range (Å)	2.5-3.5	2.6-3.5	2.8-3.5
FSC threshold	0.143	0.143	0.143
**Model Refinement**			
Refinement package	PHENIX-1.17.1-3660	PHENIX-1.17.1-3660	PHENIX-1.17.1-3660
Real or reciprocal space	Real space	Real space	Real space
Model-Map CC (mask)	0.71	0.65	0.63
Model resolution (Å)	3.33	3.44	3.54
FSC threshold	0.5	0.5	0.5
B factors (Å2, mean value)			
Protein residues	18.00/126.95/60.84	18.00/126.95/60.84	18.00/126.95/60.84
Ligands	20.00/20.00/20.00	20.00/20.00/20.00	20.00/20.00/20.00
**Model composition**			
Non-hydrogen atoms	9,986	9,989	9,990
Protein residues	1,275	1,275	1,275
R.m.s. deviations			
Bond lengths (Å)	0.002 (0)	0.002 (0)	0.002 (0)
Bond angles (°)	0.467 (8)	0.387 (0)	0.445 (3)
**Validation**			
MolProbity score	1.28	1.25	1.41
Clashscore	5.19	4.78	7.45
Rotamer outliers (%)	0.64	0.00	0.18
Ramachandran plot			
Favored (%)	98.65	99.20	99.12
Allowed (%)	1.27	0.80	0.88
Disallowed (%)	0.08	0	0
**Data availability**			
EMDB entry	EMD-35724	EMD-35725	EMD-35726
PDB entry	8IUK	8IUL	8IUM

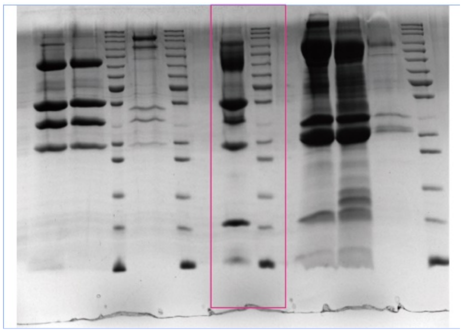

Uncropped version of the gel in Supplemental Figure 1b.

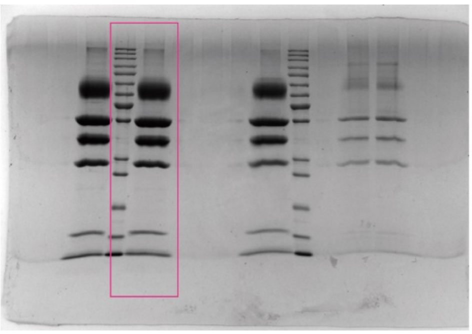

Uncropped version of the gel in Supplemental Figure 2b.

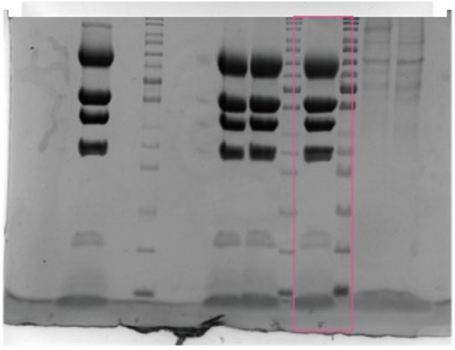

Uncropped version of the gel in Supplemental Figure 3b.
